# PaReBrick: PArallel REarrangements and BReaks identification toolkit

**DOI:** 10.1093/bioinformatics/btab691

**Published:** 2021-10-02

**Authors:** Alexey Zabelkin, Yulia Yakovleva, Olga Bochkareva, Nikita Alexeev

**Affiliations:** Computer Technologies Laboratory, ITMO University, St Petersburg 197101, Russia; Bioinformatics Institute, St Petersburg 194100, Russia; Bioinformatics Institute, St Petersburg 194100, Russia; Department of Microbiology, Faculty of Biology, Saint Petersburg State University, St Petersburg 199034, Russia; Institute of Science and Technology (IST Austria), 3400 Klosterneuburg, Austria; Computer Technologies Laboratory, ITMO University, St Petersburg 197101, Russia

## Abstract

**Motivation:**

High plasticity of bacterial genomes is provided by numerous mechanisms including horizontal gene transfer and recombination via numerous flanking repeats. Genome rearrangements such as inversions, deletions, insertions and duplications may independently occur in different strains, providing parallel adaptation or phenotypic diversity. Specifically, such rearrangements might be responsible for virulence, antibiotic resistance and antigenic variation. However, identification of such events requires laborious manual inspection and verification of phyletic pattern consistency.

**Results:**

Here, we define the term ‘parallel rearrangements’ as events that occur independently in phylogenetically distant bacterial strains and present a formalization of the problem of parallel rearrangements calling. We implement an algorithmic solution for the identification of parallel rearrangements in bacterial populations as a tool PaReBrick. The tool takes a collection of strains represented as a sequence of oriented synteny blocks and a phylogenetic tree as input data. It identifies rearrangements, tests them for consistency with a tree, and sorts the events by their parallelism score. The tool provides diagrams of the neighbors for each block of interest, allowing the detection of horizontally transferred blocks or their extra copies and the inversions in which copied blocks are involved. We demonstrated PaReBrick’s efficiency and accuracy and showed its potential to detect genome rearrangements responsible for pathogenicity and adaptation in bacterial genomes.

**Availability and implementation:**

PaReBrick is written in Python and is available on GitHub: https://github.com/ctlab/parallel-rearrangements.

**Supplementary information:**

Supplementary data are available at *Bioinformatics* online.

## 1 Introduction

Large-scale deletions and inversions affect different levels of chromosome organization, and are mostly deleterious (Darling *et al.*, 2008; Repar and Warnecke, 2017). Nevertheless, beneficial rearrangements are also known, such as those that lead to the acquisition of new function, phenotype switching or rapid genome reduction (Brandis and Hughes, 2020).

Such beneficial rearrangements may occur independently in different strains, which leads to instances of parallel adaptation to new environments or phenotypic diversity (Seferbekova *et al.*, 2021). Phenotypic diversity in clonal populations is shaped by a mechanism of reversible alternation between genetic states known as phase variation (Trzilova and Tamayo, 2021). Such reversible large-scale DNA inversions affecting complex bacterial phenotypes including antibiotic resistance were found in many human pathogens and were associated with persistent infections (Guérillot *et al.*, 2019; Irvine *et al.*, 2019). Moreover, antigenic variation has been described in the human pathogen *Streptococcus pneumoniae* (Shelyakin *et al.*, 2019; Slager *et al.*, 2018), which targets reversible inversions affecting surface antigens, encoded by *phtB and phtD* genes, previously considered to be good vaccine candidates. Therefore, the identification of phase variation in pathogenic bacteria can enhance the understanding of the molecular basis of pathogenicity and serve as a useful tool for detection of specific genes of interest.

Presently, the identification of parallel rearrangements providing adaptation or phenotype switching is performed by laborious manual inspection of complex phylogenetic and genomic data (Bochkareva *et al.*, 2018; Seferbekova *et al.*, 2021; Shelyakin *et al.*, 2019). The pipelines used in such studies involve the construction of phylogenetic trees, identification of synteny blocks, and in-house scripting to identify parallel synteny block inversions and deletions on the phylogeny. However, to our knowledge, there are no tools that allow for consistent and statistically rigorous analysis of synteny blocks on precomputed phylogenies.

**Fig. 1.**
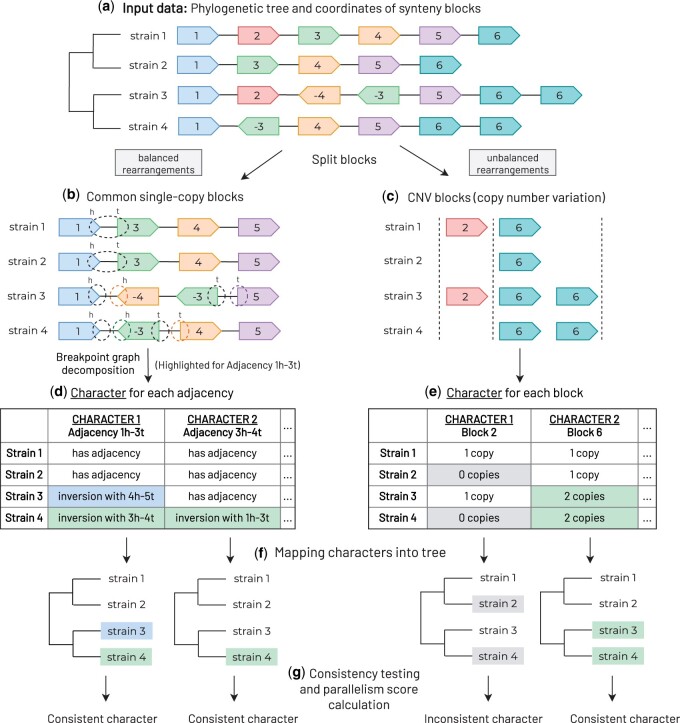
The PaReBrick pipeline: (**a**) PaReBrick takes a collection of strains and a phylogenetic tree as input data. Each strain is represented as a sequence of oriented synteny blocks, and corresponds to a leaf of the tree. The tool splits the blocks to (**b**) common single copy and (**c**) CNV block content. (**d**, **e**) For each character, the tool assigns a character state to each strain and (**f**) maps characters into the tree. (**g**) For each character, the tool tests if it is consistent with the tree; if not, it claims it is parallel and computes its parallelism score

Here, we develop a strategy for computational prediction of parallel rearrangements and analysis of genomic repeats. Our method, called PaReBrick, identifies and visualizes parallel rearrangements in bacterial genomes (Fig. 1). We characterize PaReBrick’s efficiency and accuracy and show its potential to detect genome rearrangements responsible for pathogenicity and adaptation in bacterial genomes.

## 2 Approach

### 2.1 What is parallel rearrangement?

**Fig. 2.**
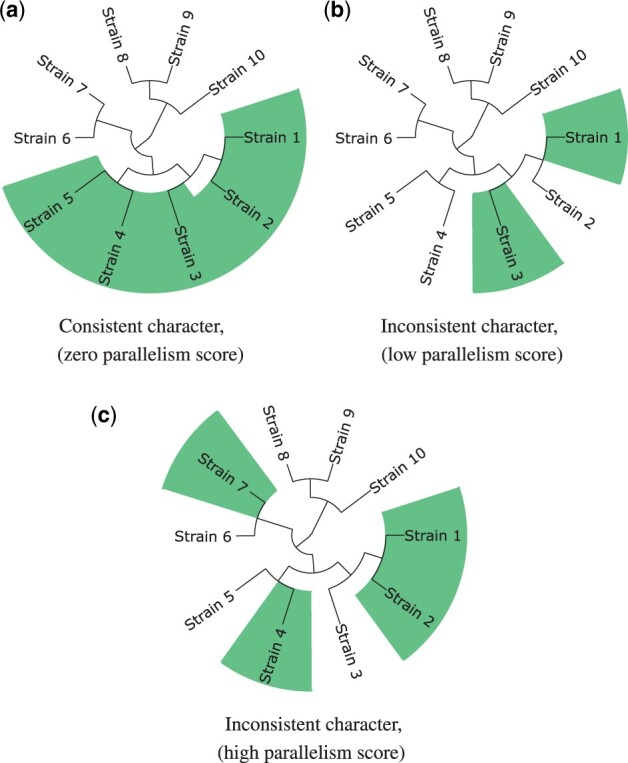
Examples of characters. Phylogenetic tree’s leaves are colored to reflect states of the characters. (**a**) Consistent character, state was changed once in common ancestor of strains 1–5. (**b**) Inconsistent character with low parallelism score; state was changed independently two times, the inconsistency may be easily explained by error in the tree construction. For instance, if the strains 1 and 3 formed a clade and strain 2 was an outgroup, then the character would be consistent. (**c**) Inconsistent character with high parallelism score, state was changed multiple times in the tree at distant branches.

We say that an evolutionary event is consistent with a tree if we may associate it with a particular branch on a tree (Fig. 2a), otherwise we call the event parallel (Fig. 2b and c). More formally, consider a character which state was changed by the evolutionary event; the character is consistent with a tree if any two strains sharing the character state have a common ancestor with the same character state.

In this article, we analyze two classes of evolutionary events: *balanced genome rearrangements*, those that change the order of synteny blocks but do not cause deletions or duplications, and *unbalanced genome rearrangements*, those that affect block copy number. We note that several balanced genome rearrangements may operate on the fragment between the same synteny blocks. We associate a character to a set of rearrangements operating on the same fragment. For the unbalanced rearrangements, we just associate a character to each block copy number. To find parallel rearrangements, we test all the introduced characters for consistency using the Fitch’s algorithm. Note that, we consider an appearance of two (or more) parallel rearrangements just by chance extremely unlikely. Indeed, we expect that the number of potential sites which may be involved in rearrangements is order of magnitude larger than the number of observed rearrangements. This assumption can be considered as a genome rearrangement analogue of the infinite site model. So we report all the discovered parallel rearrangements since any of them may have biological meaning.

To range characters with different degrees of inconsistency, we introduce a *parallelism score* (see Section 3.2). Specifically, input data may include various types of artifacts, such as misalignments as well as genome sequencing and assembly errors, generating errors in phylogeny reconstruction. Therefore, it is useful to the user to have a degree of confidence through a parallelism score, which takes into account how often the corresponding character changed its state and how far in the tree these changes happened. For example, in Figure 2b, the rearrangement is observed independently two times, but the corresponding nodes in a tree are close to each other. In this case, the character will have a low parallelism score because inconsistency may be caused by incorrect topology of a specific tree clade, especially if the branches are short. In contrast, in Figure 2c, the rearrangement occurred independently in several distant nodes, so there is higher confidence that this is an actual parallel event.

### 2.2 Pipeline description

PaReBrick takes a collection of strains and a phylogenetic tree as input data. Each strain is represented as a sequence of oriented synteny blocks, and corresponds to a leaf of the tree. To test rearrangements for being parallel, we first associate them to characters which are based on synteny blocks data. To do so, we split all blocks (Fig. 1a) into common single-copy blocks (Fig. 1b, balanced case) and blocks with CNV (copy number variations) (Fig. 1c, unbalanced case). Then, we construct characters for balanced and unbalanced rearrangements independently (Fig. 1d and e) (see Section 3.1). We test obtained characters for consistency with a tree (Fig. 1g) by mapping those characters into phylogenetic tree (Fig. 1f) and range the inconsistent characters according to the parallelism score (see Section 3.2).

## 3 Materials and methods

### 3.1 Characters assignment

**Characters for unbalanced rearrangements.** To analyze indels and duplications, we consider all blocks which are present in different copy numbers in the strains (CNV blocks). We associate a character with each block *B* and assign to each strain a character state equal to the number of copies of *B* presented in this strain (Fig. 2c).

Genome rearrangements may occur on different evolutionary scales and overlap that may lead to block linkage evolution. For example, if a long fragment was inserted into some ancestral genome and after that its different pieces were deleted in several descendants, then the resulting blocks would have similar but not identical occurrence patterns. To take this phenomenon into account, PaReBrick automatically clusters the blocks. For clustering, we define the proximity measure between blocks as a combination of the similarity of their occurrence patterns and the typical genomic distance between them in the strains (see Supplementary Appendix D for more details). This step also improves readability of the results and reduces output size.

**Characters for balanced rearrangements.** To analyze balanced rearrangements, we concentrate on common single-copy block content, that is, we consider only those blocks present in each strain exactly once.

We represent each strains’ genome as a circular sequence of synteny blocks. Each block (say, block 1) is represented by its tail (1*t*) and head (1*h*). Consecutive blocks (say, blocks 1 and 2) are linked by an *adjacency* (1h−2t) (Fig. 2b). We say that an adjacency is *consensus* if it is presented in the majority of strains.

We associate a character with each consensus adjacency. To assign a character state to a strain we check this adjacency’s status in the strain (if it is presented or broken). In other words, for each consensus adjacency, we construct a character and associate with it all rearrangements that affect this adjacency.

More formally, we run the following procedure. We construct a *breakpoint (multi)graph* for the collection of strains’ genomes. The vertices of the graph correspond to the ends of synteny blocks. For each strain *S*, we add all its adjacencies to the graph as edges with label *S*. So, if we have *m* strains on *n* common blocks, the graph would have 2*n* vertices and *mn* edges (see Supplementary Fig. S1d).

We note that an inversion between two strains, say *P and Q*, corresponds to a 4-cycle in a breakpoint graph. The 4-cycle consists of two *P*-adjacencies and two *Q*-adjacencies (see Supplementary Fig. S1). The 2-cycles in the breakpoint graph correspond to the case then both strains have the same adjacency, and other configurations corresponds to *breaks—*more complex rearrangements or series of inversions.

We associate a character with each consensus multi-edge (*u*, *v*) and assign the character state to each strain *S* according to the classification below (see Supplementary Appendix A.2 for examples):

1. The strain **has the adjacency** (*u*, *v*);
2. The strain does not have the adjacency (*u*, *v*). We distinguish two options here:
  - Character state of *S* is classified as an **inversion** (of *u–v*) **with *w–z*** if there is a 4-cycle u,v,w,z in the breakpoint graph with the following properties. The vertices *w and z* are adjacent to the vertices *u and v* (respectively) in the strain *S*, and they are adjacent to each other in some strain *P* which is having adjacency (*u*, *v*).
  - Character state of *S* is classified as a **break** (of *u–v*) if there is no such a 4-cycle. In this case the breakage is a result of multiple rearrangements and cannot be explained by a single inversion.

### 3.2 Consistency testing and parallelism score

We test each character for consistency with the phylogenetic tree with the standard Fitch’s algorithm (Fitch, 1971). If a character is consistent with the tree, we claim that there is no parallel rearrangement associated with it. Otherwise, we claim that there is a parallel event corresponding to the character. To range the inconsistent characters, we compute their *parallelism scores* which take into account the number and the phylogenetic positions of the state changes.

To do this, first of all we reconstruct for each character *c* the character states in the inner nodes with a modified version of maximum parsimony search algorithm (see Supplementary Appendix B). For inconsistent character, the reconstruction of inner nodes’ states can be not unique, but the algorithm finds a deterministic and reasonable solution. Note that, we do it independently for each character to compute the parallelism score only, and we reconstruct neither the structure of ancestral genomes nor evolutionary scenarios (which is not our goal).

After that, for each state *s* of character *c* we find the set of all the vertices *V_s_* on a tree where it appeared: $V_{s}=\{v|\text{state}(v)=s\&\;\text{state}(\text{ancestor}(v))\neq s\}$.

Assume that some *character state is inconsistent* with a tree if $|V_{s}|>1$ (see Supplementary Appendix C for examples). We note that the inconsistent character is a character with at least one inconsistent state.

**Fig. 3.**
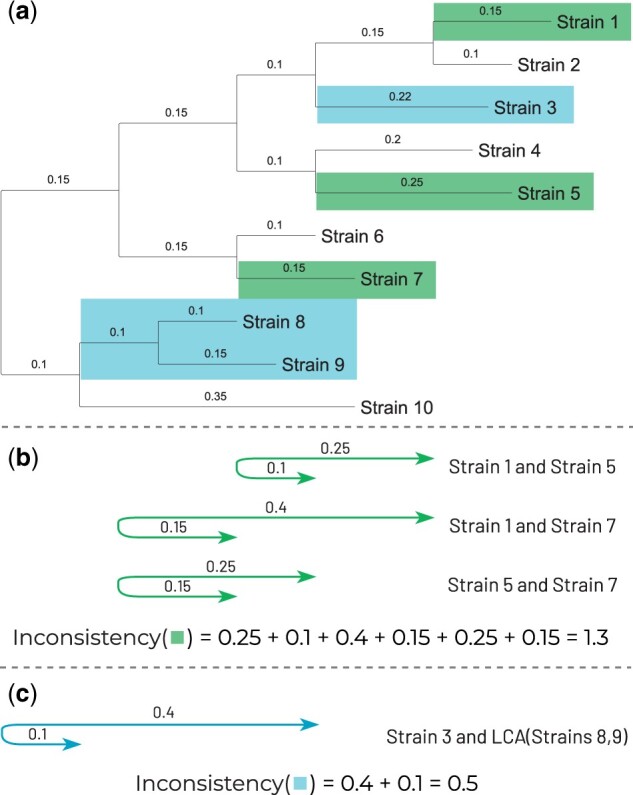
Example of calculation of parallelism score of a character. (**a**) The phylogenetic tree is colored to reflect states of the character (shown in blue, green and white). For this character, white state is consistent, while blue and green states are inconsistent and contribute to parallelism score. (**b, c**) Inconsistency of a state is calculated as a sum of the distances between all pairs of its independent appearances. Parallelism score of a character is equal to a sum of inconsistencies of its states: PS(c) = 1.3 + 0.5 = 1.8

We define the parallelism score of a character *c* as a sum of inconsistencies for all states:

$$PS(c)=\sum_{s:\;\text{state}\;\text{of}\;c}\text{Inconsistency}(s),$$

where state inconsistency is calculated as the sum of the distances between all pairs of its independent appearances:

$$\text{Inconsistency}(s)=\sum_{u,v\in V_{s}}d(\text{ancestor}(u),\text{ancestor}(v)),$$

where *d*(*u*, *v*)—is distance between nodes on a phylogenetic tree (see example on Fig. 3).

We also introduce the *break score* to rank the rearrangements resulted from multiple breakages, see Supplementary Appendix C.2 for details.

### 3.3 Neighborhood visualization

**Fig. 4.**
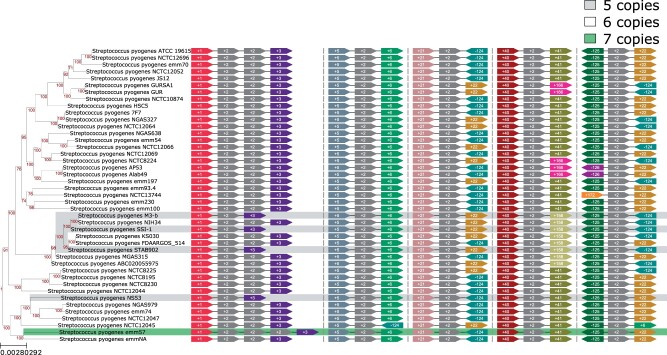
The genomic context of block #2, containing the rRNA gene operon. For each strain and each copy of the block, the upstream and the downstream neighboring block are shown. It revealed a variation of number of tandem copies in the first loci and a parallel inversion between copies in the third and the fifth loci. For better visibility, the subtree is shown

As various molecular mechanisms might be responsible for variations in block content, we provide a diagram of the neighbors for each block in each genome where it is present (see Fig. 4). For easier comparison of their context in different genomes, all blocks’ copies are rotated on the same side and grouped into columns based on similarity of their neighbors. If tandem copies of a block are present, all copies are visualized. This visualization aims for the best readability of blocks’ context data and therefore does not reflect the order of the loci in genomes nor blocks’ length. Meanwhile, it allows to detect horizontally transferred blocks and to distinguish between copies of a block. For multi-copied blocks, this visualization reveals inversions in which these repeats are involved.

## 4 Results

### 4.1 Data preprocessing

**Phylogenetic tree.** There are many implemented approaches to construct phylogenetic trees which are usually based on concatenated alignment of homologous genes. In our study, we use the PanACoTA pipeline (Perrin and Rocha, 2021) which includes all steps for phylogenetic tree construction including genome annotation and orthologs detection. Thus, intermediate results can be further used for biological annotation and interpretation of parallel rearrangements.

**Synteny blocks.** We understand by *synteny blocks* a decomposition of genomes into non-overlapping highly conserved segments. Synteny blocks can be defined on different scales depending on the field of study, and the scale is usually controlled by the threshold of minimal block length. Synteny blocks are often constructed based on *seeds* (also called *anchors*), with most methods using the ‘seed-and-extend’ approach. Homologous genes, locally collinear blocks or any multiple whole-genome alignment results can be used as seeds. In our study, we use an efficient multiple whole-genome alignment tool SibeliaZ (Minkin and Medvedev, 2020) to obtain locally collinear blocks and its submodule maf2synteny (Kolmogorov *et al.*, 2014) to construct synteny blocks.

### 4.2 Application to *Streptococcus* genomes

First, we applied the PaReBrick tool to complete genomes of *S.pneumoniae* as an antigenic variation via large-scale inversion between repeats in *PhtB and PhtD* genes has been previously described in this species (Shelyakin *et al.*, 2019; Slager *et al.*, 2018). The complete assemblies of *S.pyogenes* were downloaded from the NCBI RefSeq database, all plasmids were excluded. Indeed, our tool assigned the highest parallelism scores to the adjacencies affected by this inversion (Supplementary Fig. S6). PaReBrick detected nine strains across the phylogenetic tree that have this fragment inverted, only five of them were previously identified in (Shelyakin *et al.*, 2019). Moreover, PaReBrick detected additional rearrangement events affecting these adjacencies in several strains.

Then, we used the PaReBrick tool to detect and classify parallel rearrangements in 219 *S.pyogenes* genomes (see Supplementary Appendix E in Supplementary Material) downloaded from the NCBI RefSeq database. The whole project including the input and output of the tool is available at the Github repository: github.com/ctlab/parallel-rearrangements.

**Fig. 5.**
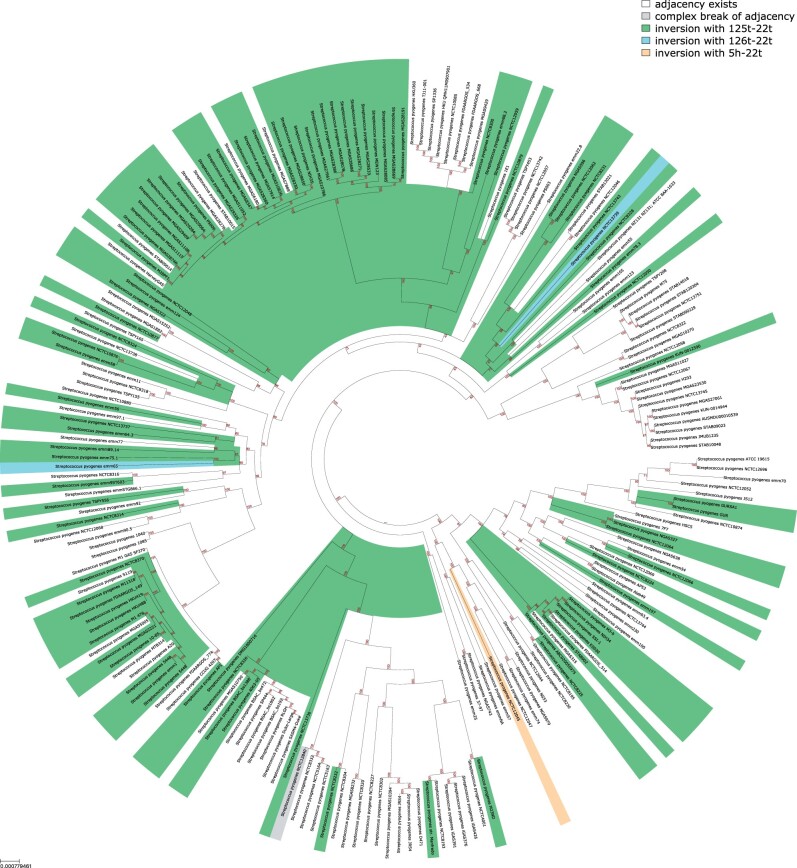
The adjacency with the highest parallelism score in *S.pyogenes*. The tree is colored to reflect the states of the character assigned to 124h–21h (the first line in Table 1). White state corresponds to the presence of this adjacency, while green, blue and orange reflect different inversions that affect this adjacency. While orange state is strain-specific and unique, green and blue states are inconsistent. Green state reflects the inversion of 1.4 Mb fragment between 124h–21h and 125t–22t adjacencies containing rRNA gene operons

In total, we analyzed 217 synteny blocks, 125 of them present exactly once in each strain. Among six adjacencies involved in parallel rearrangements (Table 1), the highest parallelism scores were assigned to the adjacencies 124h–21h and 125t–22t affected by 1.4 Mb parallel inversion occurred 51 times across the tree (Fig. 5). In all strains, these adjacencies contain the multi-copied block with a *rRNA* operon, indicating its involvement in the recombination. The mean length of adjacencies (7–15 kb) is consistent with the operon length, which also validates the observation. These data closely resemble previously described inversions that have been shown to affect the underlying phenotype, including inversion of the fragment between rRNA genes in *Pseudomonas aeruginosa* affecting resistance to oxidative stress, central metabolism and virulence (Irvine *et al.*, 2019). Thus, these data indicate that it is feasible to computationally detect parallel genomic events in closely related strains, putative associated with phase variation in bacterial populations.

**Table 1.** Summary table for the adjacencies in *S.pyogenes* affected by *balanced rearrangements*, only characters with *parallelism score* more than zero are shown

Adjacency	Mean break length (nucleotide)	Parallelism score
124h–21h	7894	6.57
125t–22t	16751	6.37
101h–103t	30744	0.013
51h–52t	20481	0.013
135h–136t	1665	0.0002
16h–17t	3363	0.0002

*Note*: The highest parallelism scores were assigned to the adjacencies 124h–21h and 125t–22t (two first lines in the table) that are affected by the same parallel inversion. The parallelism score assigned to 124h–21h is slightly higher than the score assigned to 125t–22t since there are extra events affecting the first adjacency but not the second one (Fig. 5).

**Fig. 6.**
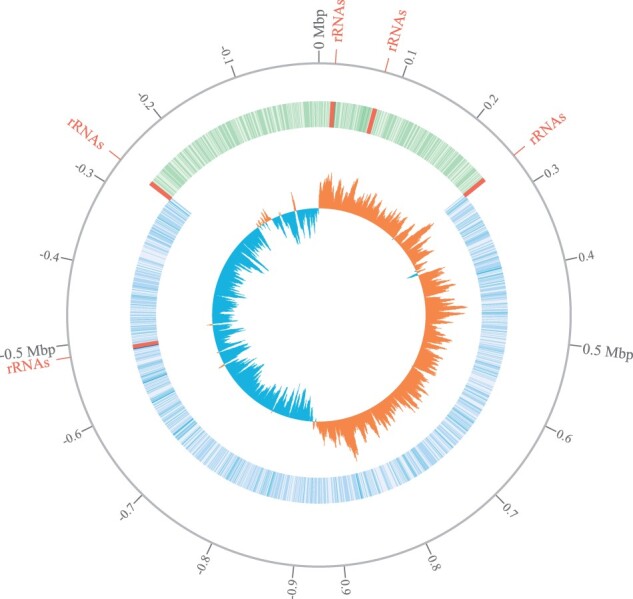
Relative positions of five loci containing rRNA gene operons in chromosomes of *S.pyogenes*. The detected parallel inversion typically occurs between a pair of symmetric repeats: for the presented *S.pyogenes* strain BSAC_bs472 the distances from the origin to these repeats are 258 996 and 265 532 (based on rRNA block coordinates); and for 50% of all analyzed strains the corresponding repeats are located even more symmetrically. The inner blue-orange circle shows GC-skew, 0 Mb corresponds to origin of replication (*ori*). The second circle represents the annotated genes, the green fraction of the chromosome is involved in parallel inversion. The borders of the inversion are formed by inverted copies of the rRNA gene operon located at the same distance from the ori

For 65 out of 217 blocks, we revealed parallel insertions, deletions and multiplications (Table 2); the highest scores were assigned to phage insertions (Supplementary Fig. S7). Visualization of genome context for these blocks revealed their independent acquisition by different *S.pyogenes* lineages. In some strains, these insertions occurred two or three times in different loci (Supplementary Fig. S8). The block containing the *rRNA* operon also has high parallelism score. Indeed, while *S.pyogenes* genomes contain six copies of the block in five different loci, a few strains lost one of the tandem copies or gained up to four copies of the operon. (Fig. 4, Supplementary Figs S9 and S10). Visualization of blocks’ locations within the genomes revealed that the parallel inversion typically occurred between a pair of repeats placed symmetrically across the origin (Fig. 6).

**Table 2.** Summary table for the blocks in *S.pyogenes* affected by *unbalanced rearrangements*, only the first seven lines are shown

Block	Cluster	Parallelism score	Mean block copies	Annotation^a^
166	0	6.01	0.38	Prophage
159	1	4.65	0.35	Prophage
157	2	4.01	0.43	Prophage
156	2	3.46	0.36	Prophage
155	2	3.19	0.36	Prophage
172	3	2.15	0.11	Prophage
2	4	1.94	5.92	RNA gene operon

*Note*: The blocks with the highest parallelism scores contain phage insertions; blocks #155, 156, 157 are assigned to the same cluster.

a Annotation was performed manually.

### 4.3 Running time

Our pipeline is based on a sequence of polynomial algorithms, and its complexity depends on the number of strains *s* and the number of blocks *b*. The step of constructing characters for unbalanced rearrangements has complexity *O*(*s*) for each block. Testing a character for consistency with a tree has complexity *O*(*s*) as well. The overall complexity of the clustering step is $O(b^{2}(b+s))$ for building distance matrices and for hierarchical clustering itself. The step of constructing characters for balanced rearrangements takes *O*(*s*) for each adjacency. The total number of adjacencies is in between the number of blocks *b* (if all strains have same block order) and its square *b*^2^, but does not exceed *bs*.

In practice, this means that for the *S.pyogenes* dataset with 219 strains, the whole process takes 54 seconds on a laptop with Apple M1 CPU, and the trees’ rendering consumes the majority of the computational time, 36 of 54 seconds.

## 5 Discussion

Modern sequencing technology produces massive amounts of genomic data, providing exceptional opportunities to investigate whole-genome organization and interactions of different components (English *et al.*, 2012; Madoui *et al.*, 2015). Several strategies are widely used for assembly validation such as long read (re)sequencing and PCR contiguity verification. Nevertheless, some of the detected rearrangements may be caused by inaccuracies in gap closure procedures. In turn, if the reference genome was used for gap resolving, some strain-specific genome rearrangements might be missed. Thus, for particular computational observations, further experimental validation may be required.

Genomic repeats of different nature may play the role of substrate for recombination. Recent studies have sparked a renewed interest in large-scale phase variation, as it may affect complex bacterial phenotypes and modulate expression of a set of genes (Trzilova and Tamayo, 2021). Pathogenic bacterial species use this strategy for ensuring survival (Huang *et al.*, 2020). Phase variation might be responsible for chronic infections, providing multi-virulence, antibiotic resistance and antigenic variation (Guérillot *et al.*, 2019; Irvine *et al.*, 2019; Slager *et al.*, 2018). While particular cases are described, reversible large-scale inversions have been not investigated systematically. The PaReBrick tool allows for computational identification of phase variation through analysis of parallel inversion in closely related strains. Systematization and verification of the observed cases is the key to understanding new molecular mechanisms in pathogens.

## 6 Conclusion

The PaReBrick tool has great potential to allow researchers to address wider research questions in evolutionary, molecular and medical fields. The approach might be used for the study of rapid emergence of new bacterial phenotypes, understanding the molecular basis of antibiotic resistance mechanisms and formation of small colony variants, and the study of the selective forces in genomic evolution underlying complex phenotypes. The application of this approach and the concomitant understanding of connections between detected genome rearrangements and medically relevant phenotypes may contribute to the efficient development of drugs and vaccines.

## Supplementary Material

btab691_Supplementary_Data

## References

[btab691-B1] Bochkareva O.O. et al (2018) Genome rearrangements and selection in multi-chromosome bacteria *Burkholderia* spp. BMC Genomics, 19, 965. 30587126 10.1186/s12864-018-5245-1 PMC6307245

[btab691-B2] Brandis G. , Hughes D. (2020) The snap hypothesis: chromosomal rearrangements could emerge from positive selection during niche adaptation. PLoS Genet., 16, e1008615. 32130223 10.1371/journal.pgen.1008615 PMC7055797

[btab691-B3] Darling A. et al (2008) Dynamics of genome rearrangement in bacterial populations. PLoS Genet., 4, e1000128. 18650965 10.1371/journal.pgen.1000128 PMC2483231

[btab691-B4] English A.C. et al (2012) Mind the gap: upgrading genomes with pacific biosciences rs long-read sequencing technology. PLoS One, 7, e47768. 23185243 10.1371/journal.pone.0047768 PMC3504050

[btab691-B5] Fitch W.M. (1971) Toward defining the course of evolution: minimum change for a specific tree topology. Syst. Zool., 20, 406.

[btab691-B6] Guérillot R. et al (2019) Unstable chromosome rearrangements in *Staphylococcus aureus* cause phenotype switching associated with persistent infections. Proc. Natl. Acad. Sci. USA, 116, 20135–20140. 31527262 10.1073/pnas.1904861116 PMC6778178

[btab691-B7] Huang X. et al (2020) Prevalence of phase variable epigenetic invertons among host-associated bacteria. Nucleic Acids Res., 48, 11468–11485. 33119758 10.1093/nar/gkaa907 PMC7672463

[btab691-B8] Irvine S. et al (2019) Genomic and transcriptomic characterization of *Pseudomonas aeruginosa* small colony variants derived from a chronic infection model. Microb. Genomics, 5, e000262. 10.1099/mgen.0.000262 PMC6521587 30920365

[btab691-B9] Kolmogorov M. et al (2014) Ragout–a reference-assisted assembly tool for bacterial genomes. Bioinformatics, 30, i302–i309. 24931998 10.1093/bioinformatics/btu280 PMC4058940

[btab691-B10] Madoui M.A. et al (2015) Genome assembly using nanopore-guided long and error-free DNA reads. BMC Genomics, 16, 327. 25927464 10.1186/s12864-015-1519-z PMC4460631

[btab691-B11] Minkin I. , Medvedev P. (2020) Scalable multiple whole-genome alignment and locally collinear block construction with SibeliaZ. Nat. Commun., 11, 1–11. 33303762 10.1038/s41467-020-19777-8 PMC7728760

[btab691-B12] Perrin A. , Rocha E.P.C. (2021) PanACoTA: a modular tool for massive microbial comparative genomics. NAR Genomics Bioinf., 3, lqaa106. 10.1093/nargab/lqaa106 PMC7803007 33575648

[btab691-B13] Repar J. , Warnecke T. (2017) Non-random inversion landscapes in prokaryotic genomes are shaped by heterogeneous selection pressures. Mol. Biol. Evol., 34, 1902–1911. 28407093 10.1093/molbev/msx127 PMC5850607

[btab691-B14] Seferbekova Z. et al (2021) High rates of genome rearrangements and pathogenicity of *Shigella* spp. Front. Microbiol., 12, 628622. 33912145 10.3389/fmicb.2021.628622 PMC8072062

[btab691-B15] Shelyakin P.V. et al (2019) Micro-evolution of three *Streptococcus* species: selection, antigenic variation, and horizontal gene inflow. BMC Evol. Biol., 19, 83. 30917781 10.1186/s12862-019-1403-6 PMC6437910

[btab691-B16] Slager J. et al (2018) Deep genome annotation of the opportunistic human pathogen *Streptococcus pneumoniae* d39. Nucleic Acids Res., 46, 9971–9989. 30107613 10.1093/nar/gky725 PMC6212727

[btab691-B17] Trzilova D. , Tamayo R. (2021) Site-specific recombination – how simple DNA inversions produce complex phenotypic heterogeneity in bacterial populations. Trends Genet., 37, 59–72. 33008627 10.1016/j.tig.2020.09.004 PMC7755746

