# Supplementary material for: PaReBrick: PArallel REarrangements and BReaks identification toolkit
Source: Bioinformatics. 2021 Oct 2;38(2):357–63. doi: 10.1093/bioinformatics/btab691 (PMC8723149; doi:10.1093/bioinformatics/btab691)
Supplement: btab691_Supplementary_Data [file btab691_supplementary_data.pdf]

Supplementary Data for PaReBrick: Parallel Rearrangements and Breaks identification toolkit

Alexey Zabelkin, Yulia Yakovleva,
Olga O. Bochkareva and Nikita Alexeev

Appendix A Characters for Balanced Rearrangements Assignment

Characters assignment for balanced rearrangements is based on decomposition of breakpoint graph. In this section we describe concept of breakpoint graph and proposed algorithm in detatils.

Appendix A.1 Breakpoint Graph

We assume that the genome consists of a ordered sequence of synteny blocks. The genome can be represented as a graph, the vertices of which are the starts (t for tail) and ends (h for head) of the blocks. And the graph consists of directed block edges encoding blocks and their boundaries (shown by thick and directed edges in Fig. S1a, S1b), as well as undirected adjacency edges, encoding adjacencies (neighborhoods) between blocks. Fig. S1a shows the genome graph of the single-chromosome cyclic genome $P = (0, 1, 2, 3, 4, 5)$, the adjacency edges are colored in blue. Fig. S1b shows the genome graph of the single-chromosome cyclic genome $Q = (0, 1, -4, -3, -2, 5)$, the adjacency edges are colored in red. Breakpoint graph $G(P, Q)$ — superposition of genomes graphs P and Q with deleted block edges (Fig. S1c).

Since this work considers more than two bacterial genomes (strains), we use multiple breakpoint graphs. This type of graph differs from the classical breakpoint graph in that it contains adjacency edges of more than two types (see Fig. S1d).

Appendix A.2 Assignment — Breakpoint Graph Decomposition Algorithm

Each inversion corresponds to a 4-cycle in the breakpoint graph. To search for such inversions, we use the following polynomial algorithm.

We consider each adjacency of the graph in a loop and construct a character and its states for each one (Fig. S2). In real-world applications, we keep only adjacencies that are presented in at least half of the strains for improving output readability. Let's consider adjacency 1h—2t as an example. Those strains that

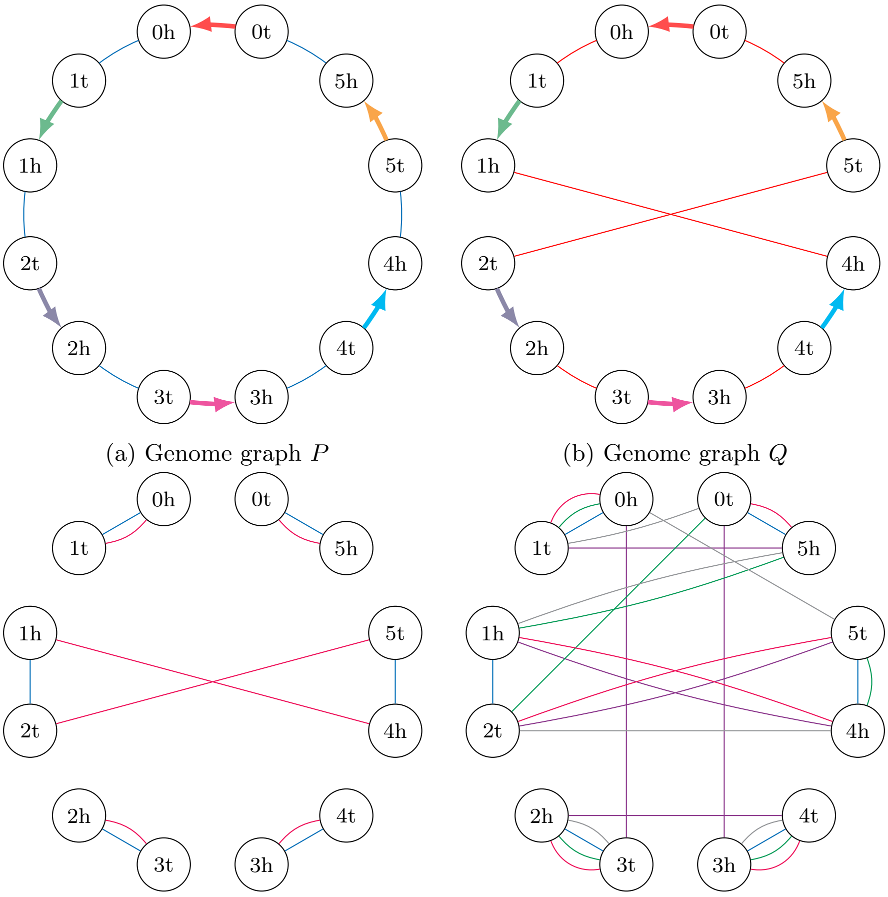

(a) Genome graph P (b) Genome graph Q

(c) Breakpoint graph $G(P, Q)$ (d) Multiple breakpoint graph for 5 strains

Figure S1: Breakpoint graphs. **(a,b)** Genome graphs P and Q which differ by inversion $1h - 2t$ with $4h - 5t$. **(c)** Breakpoint graph built for genomes P and Q, the 4-cycle corresponds to the inversion.

have this adjacency is white, the states of the character are called "Has adjacency" (e.g. Fig. S2c). Further, we look at all of all possible 4-cycles with this adjacency and each observed 4-cycle is marked as a separate character state and called " Inversion between x—y and z—v" (e.g. Fig. S2d, S2e), where (x, y) — the original edge, (z, v) — the edge with which the inversion occurred. All the remaining species have character state "Break" and marked grey due to the impossibility of explaining observed state with single inversion (e.g. Fig. S2f).

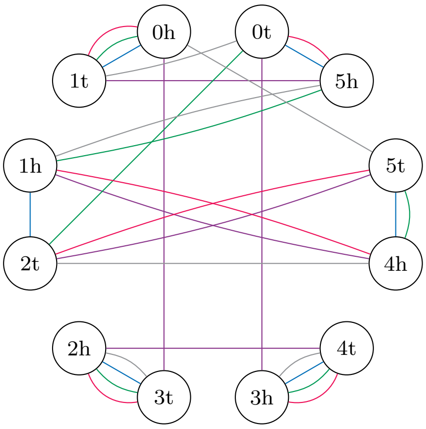

(a) Breakpoint graph example

Strain 1
Strain 2
Strain 3
Strain 4
Strain 5

(b) Correspondence between strains and their colors in a breakpoint graph

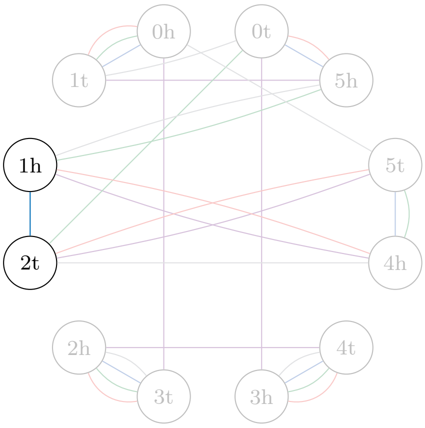

(c) “Has adjacency”, Strain 1

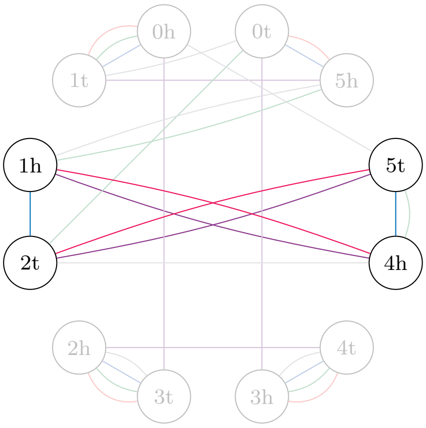

(d) “Inversion with 4h—5t”, Strain 2, Strain 3

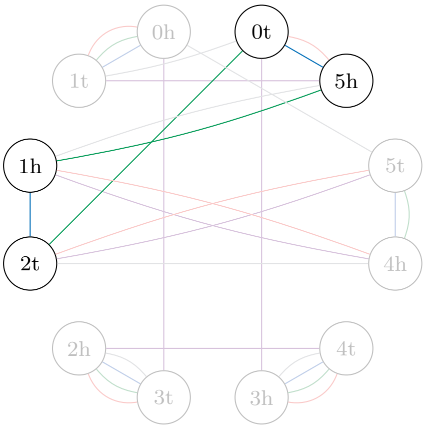

(e) “Inversion with 5h—0t”, Strain 4

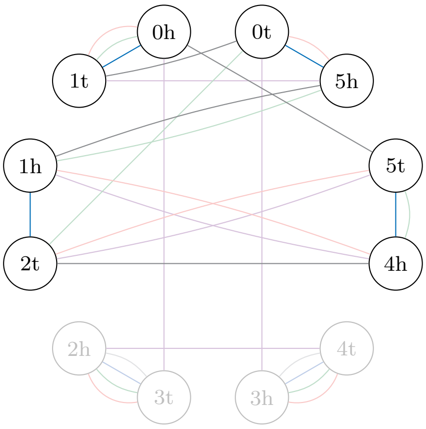

(f) “Break”, Strain 5

Figure S2: Example of character state assignment for 5 strains. Here we assume that there are more than 5 strains in the dataset, most of them have adjacency 1h-2t, but we draw only 5 strains for better figure readability. The character associated with the consensus adjacency 1h-2t has the states as written at the subfigures **c,d,e,f**.

Appendix B Reconstruction of Character State for Inner Tree Nodes

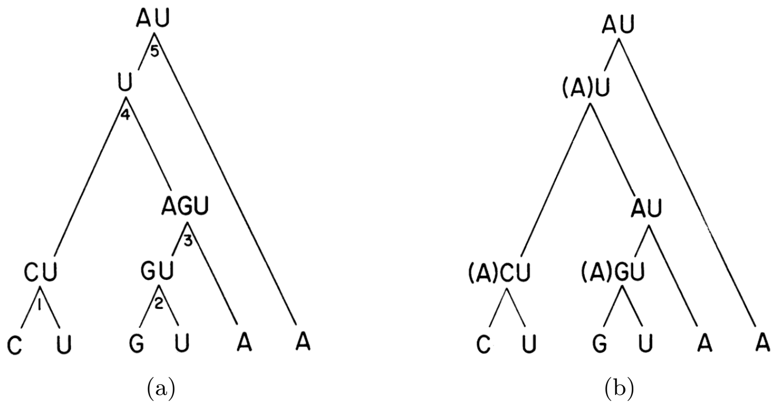

Figure S3: Reconstruction of inner states of the nucleotide sequence. (a) Preliminary reconstruction, each inner node corresponds to a set of possible states. (b) The result of the final phase of the algorithm. The nucleotides in brackets were selected (Fitch, 1971).

After constructing characters for all strains, it is necessary to test the their consistency with a tree. To solve this problem, we use a modified version of Fitch's algorithm (Fitch, 1971), and the modification is inspired by an idea of "weights" from (Erdős and Székely, 1994). Usually the algorithm is applied for reconstruction of the nucleotide sequences in inner nodes and search for an evolutionary scenario with a minimum number of substitutions (also called the maximum parsimony search algorithm).

The method can be applied to find the inner nodes' character states for any character. The important property of this algorithm is that if the character was consistent then the algorithm reconstructs the states in inner nodes in a consistent way.

The idea behind this algorithm is to traverse the tree recursively:

1. First, we go "up" along the tree from the leaves to the root. For each inner vertex we collect a set of possible states as follows: if the sets of children's states are not disjoint, we take their intersection, otherwise their union. The result of this stage of the algorithm is illustrated in Figure S3a.

2. Next, we go "down" along the tree from the root to the leaves. For each vertex, we select some state from those available in the tree and pass it down to the leaves in the following way. If the state passed from parent node belongs to the child possible states set, we choose it as a character state. Otherwise we choose a state from possible node states set. While original Fitch's algorithm chooses a character state from set at random, we use a "weight" of a state equals the number of leaves having this state. The result of this stage of the algorithm is illustrated in Figure S3b.

Appendix C Parallelism Score

Appendix C.1 Motivation for introducing parallelism score

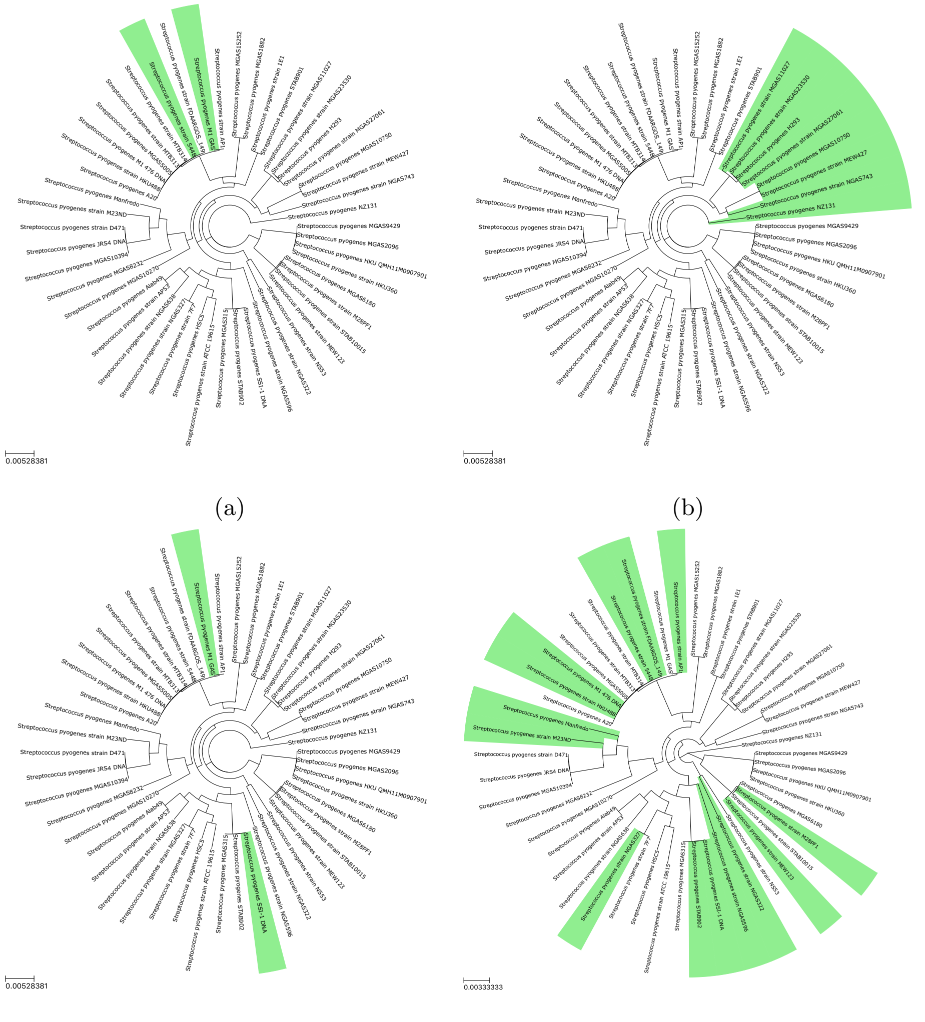

(a) (b)

(c) (d)

Figure S4: Examples of inconsistent characters in *Streptococcus pyogenes* genomes. Phylogenetic tree's leaves are colored to reflect states of the characters.

To explain the idea of characters' ranking based on their parallelism, we consider several examples of characters that are inconsistent with a phylogenetic tree (shown in Fig S4). In this section we interpret character states as colors for better readability.

- The character is diverged only in two strains, these strains are placed close to each other on short branches. This can be explained by inaccuracies in tree topology as well as independent rearrangements (Fig. S4a);

- The character is diverged in eight strains; seven of them form a clade, the eighth is an outgroup. This can be explained by two or even one event (Fig. S4b);
- The character is diverged in two strains, which are distant in the tree. This likely can be explained by two independent events at distant nodes (Fig. S4c);
- The character is diverged in fourteen strains, the pattern is mosaic. This can be explained only with several rearrangement events at distant nodes (Fig. S4d).

While all considered characters are inconsistent, the degree of such inconsistency varies. It depends on how many evolutionary events may explain this pattern, and how distant the respective nodes are in the tree.

Appendix C.2 Breaks score

To count the scores of parallel breaks we change all states except *white* (edge exist) to *grey* (any other state). Thus, the appearance of *grey* represents the break of the considered edge. We also introduce the additional metrics:

1. *Parallel break score*: Inconsistency(*grey*), parallel rearrangement score for edge breaks;
2. *Number of parallel breaks*: $|V_{grey}|$, number of independent breaks of edge.

Example The edge is broken in five strains out of ten in the tree (Fig. S5). According to the procedure for breaks, we switch all states except *white* to *grey*. In this case, appearance of *grey* represents a break while *white* indicates the edge exists.

Calculation of additional metrics for estimation of breakpoint scores:

1. *Parallel break score*: Inconsistency(grey) = 4.1, parallel rearrangement score for breaks of edge;
2. *Number of parallel breaks*: $|V_{grey}| = 5$, number of independent breaks of edge.

The PaReBrick tool uses *parallel rearrangement score* to sort the output results. As a second priority it uses *parallel breaks score* when it is applicable.

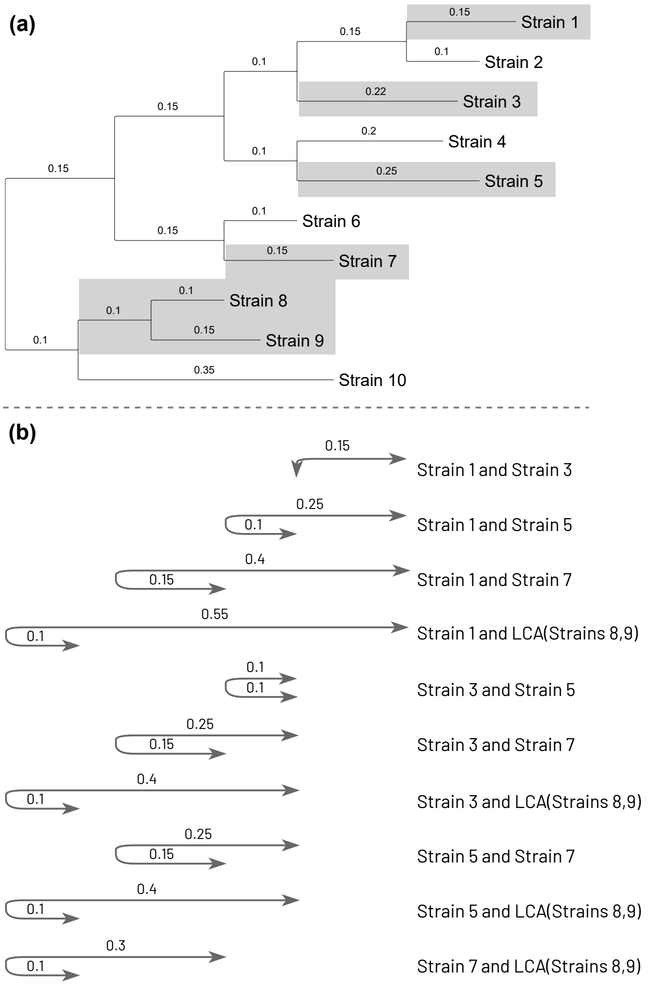

Inconsistency(■) = 0.15 + 0.25 + 0.1 + 0.4 + 0.15 + 0.55 + 0.1 + + 0.1 + 0.1 + 0.25 + 0.15 + 0.4 + 0.1 + 0.25 + 0.15 + 0.4 + 0.1 + 0.3 + 0.1 = 4.1

Figure S5: Same tree as in Fig. 3, all states except *white* changed to *grey*, with calculation example for inconsistency of *grey* which represents breaks.

Appendix D Clustering of unbalanced rearrangements by phyletic patterns

To cluster the blocks, we take into account two features:

1. *Similarity of tree patterns.* With each block b we associate a vector $v(b)$ of length n, where n is the total number of strains, in the following way: $v_k(b)$ is the copy number of the block b in the k-th strain. As a measure of dissimilarity between block patterns for blocks b_1 and b_2 we use the Manhattan distance

$$J(b_1, b_2) = \sum_{k=1}^{n} |v_k(b_1) - v_k(b_2)|.$$

2. *Distance between blocks.*

   For each pair of blocks we can calculate the minimal distance between those blocks for every strain. As a measure of distance $B(b_1, b_2)$ we take the first quartile of this distribution.

   If some blocks b_1 and b_2 don't appear together in any strain, we assign the longest genome length to $B(b_1, b_2)$.

The choice of the first quartile of the distribution of the minimal distance between all pairs of blocks as the measure of distance $B(b_1, b_2)$ is as follows. The genomic distance between blocks may differ in different strains, and we want to use some typical value as the measure. Thus, we consider blocks close to each other if they are located close to each other in at least some strains, while due to some rearrangements they may be distant in some other strains.

Further, the matrices J and B are normalized so that all their values are in the interval $[0, 1]$. Then we apply hierarchical clustering to the blocks based on a distance matrix $D = j \cdot J + b \cdot B$. Based on our experience in genomic calculations, we chose $j = 0.8, b = 0.2$ as default ones but these parameters are customizable so the users can make their own choices.

Appendix E Example of the tool application

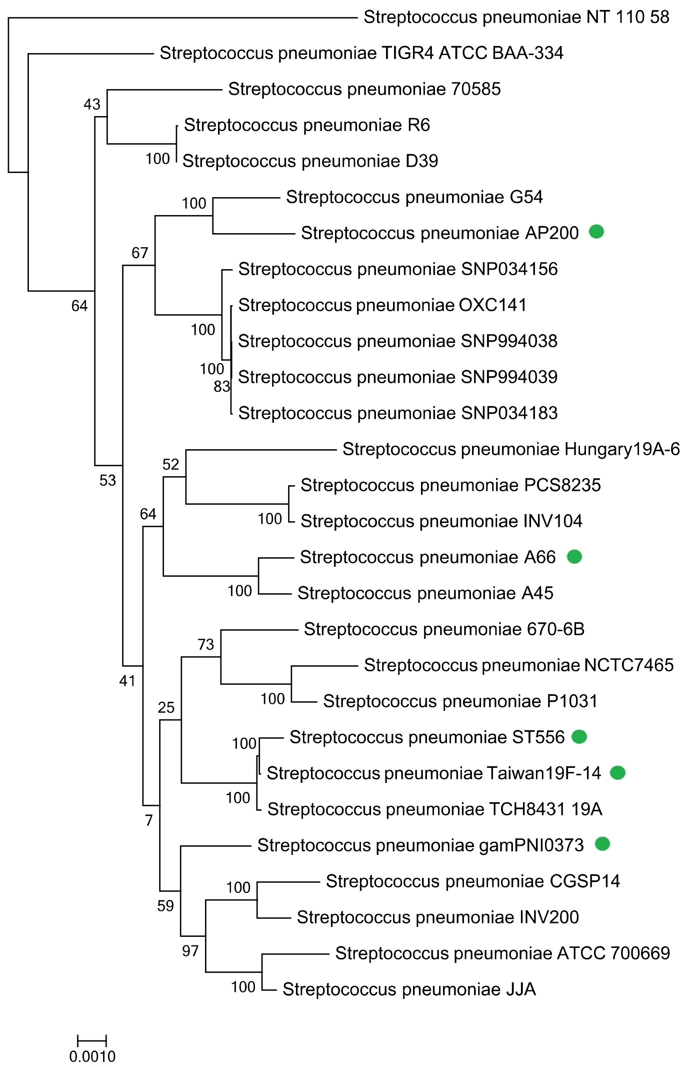

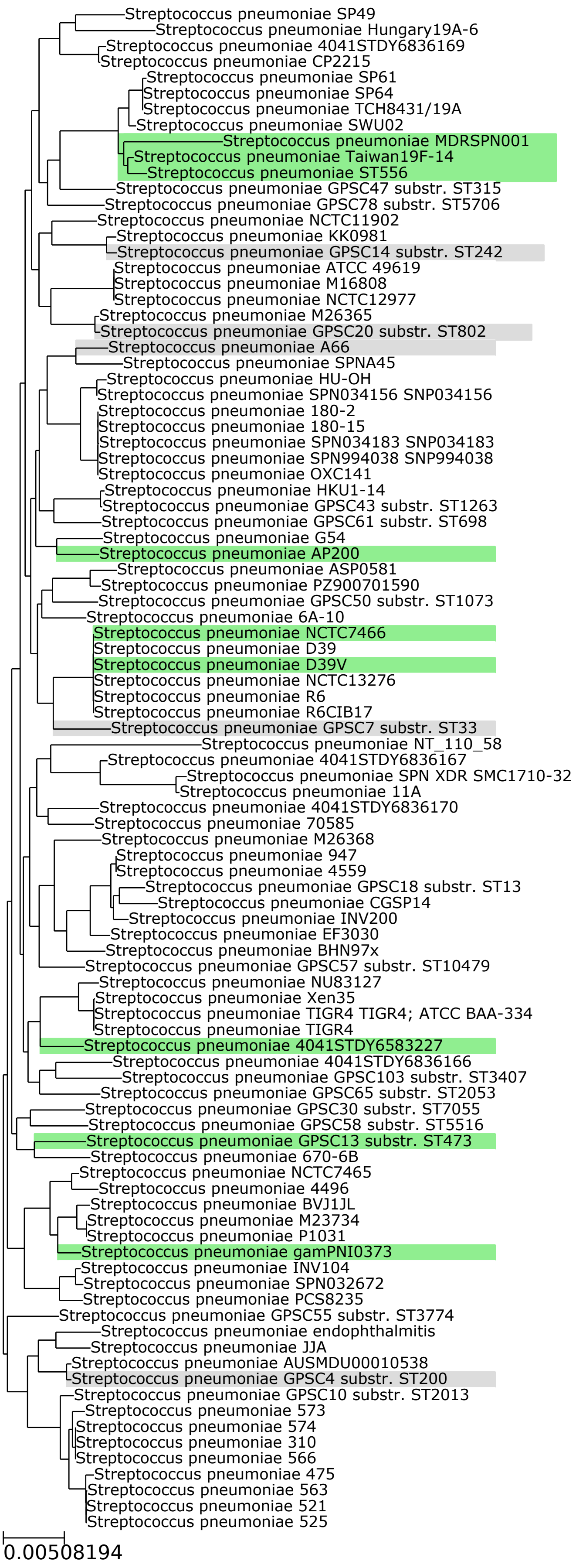

(a) the figure from the original paper (Shelyakin et al. 2019). The inversion providing antigenic variation was found in strains marked in green.

(b) the PaReBrick output. The tree is colored to reflect the states of the character assigned to one of the adjacency affected by this inversion. White state corresponds the presence of this adjacency; green state reflects the presence of inversion, grey state reflects the breakage that is a result of multiple rearrangements and can not be explained by a single inversion.

Figure S6: Detection of antigenic variation via large-scale inversion in *Streptococcus pneumoniae*, the comparison of **(a)** the original observation and **(b)** the PaReBrick output.

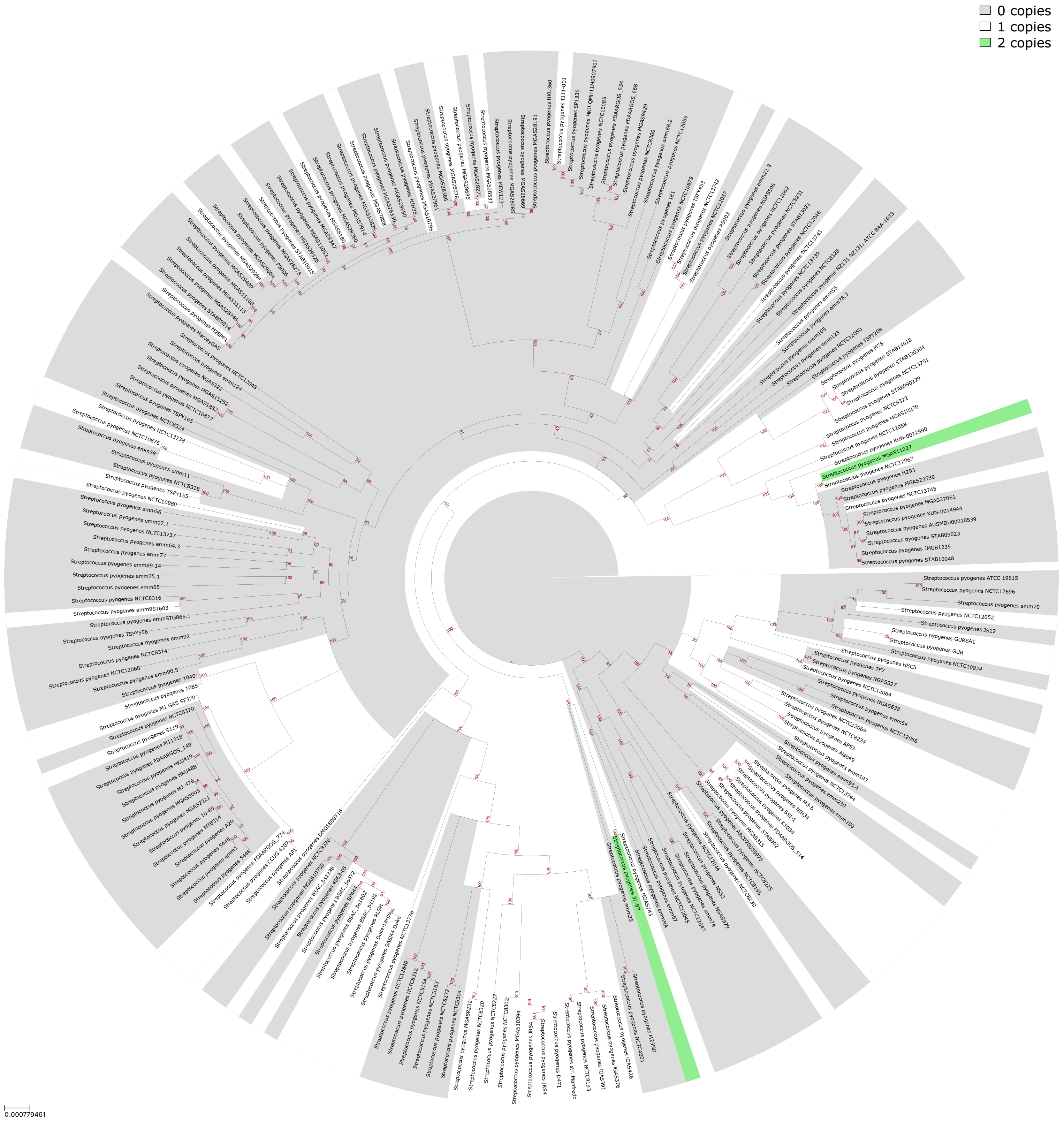

Figure S7: Copy number variation of the **block #166**. The tree is colored to reflect the states of the character assigned to copy number of this block in different strains. Grey state reflects the absence of this block, white and green states reflect the presence of one and two copies of this block correspondingly. The block consists of prophage.

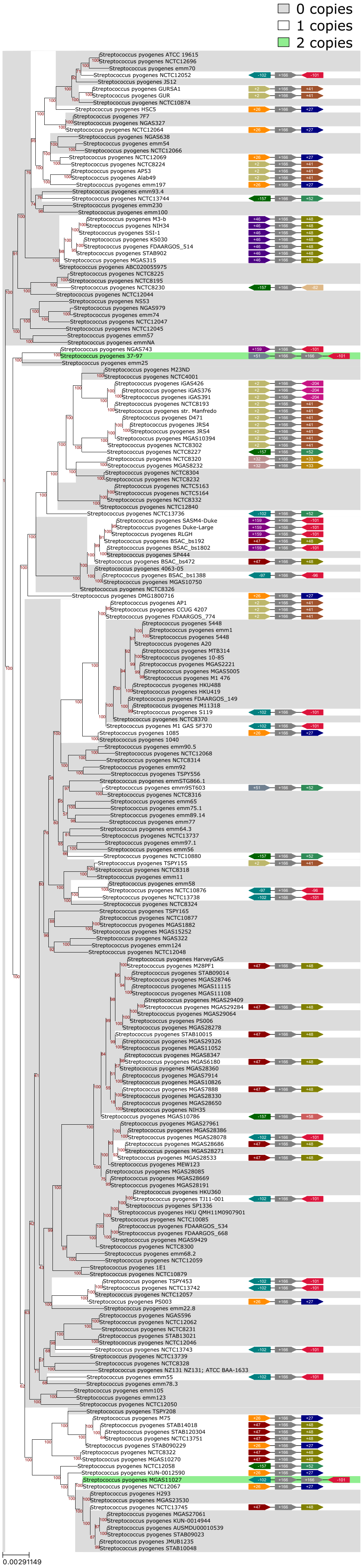

Figure S8: Genomic context of **block #166**. For each strain and each copy of the block, the upstream and the downstream neighbouring blocks are shown, revealing independent acquisition of the block by different strains.

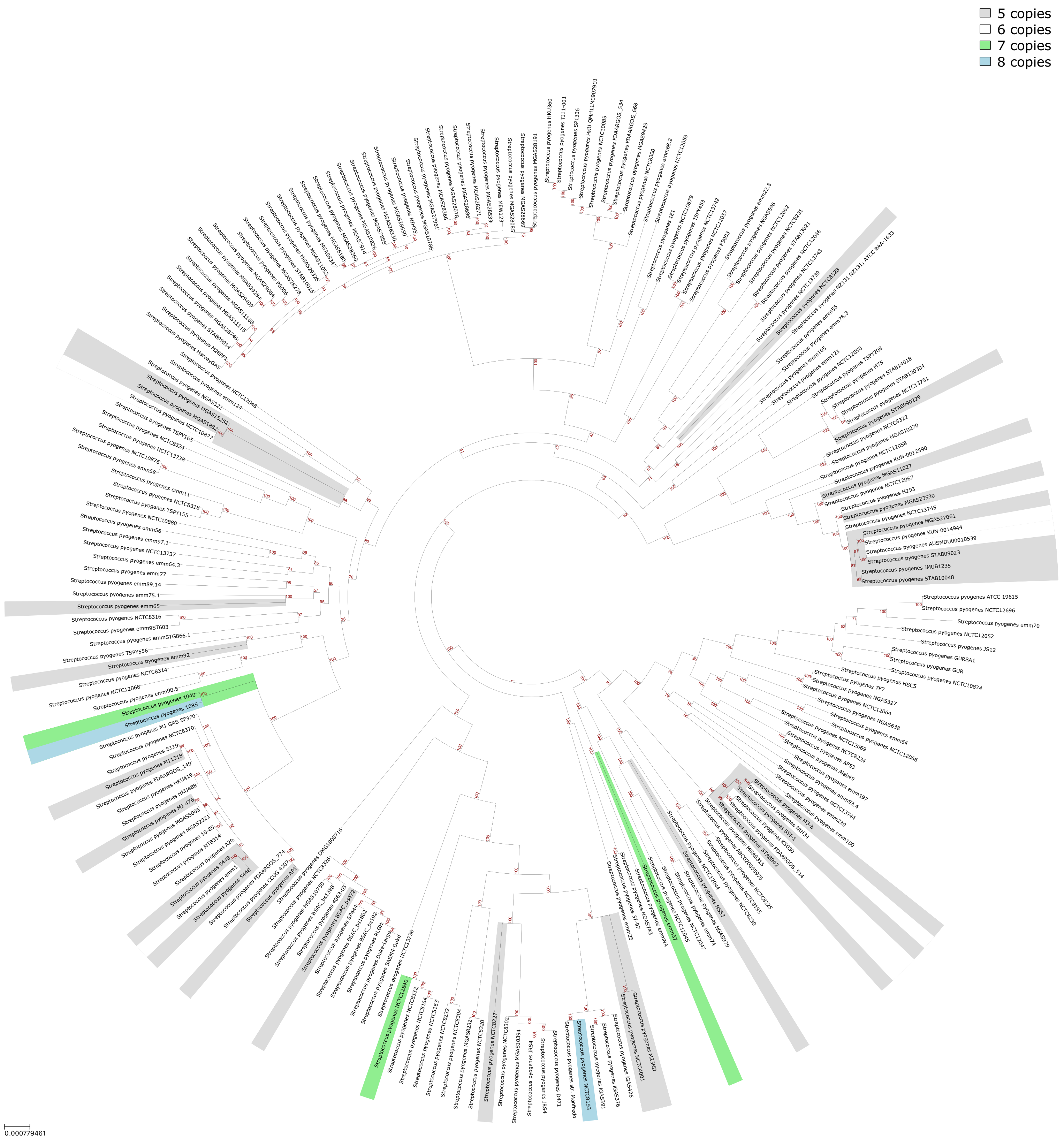

Figure S9: Copy number variation of the **block #2**. The tree is colored to reflect the states of the character assigned to copy number of this block in different strains. Grey , white, green, and blue states reflect the presence of five, six, seven, and eight copies of this block correspondingly. The block consists of ribosomal RNA (rRNA) genes.

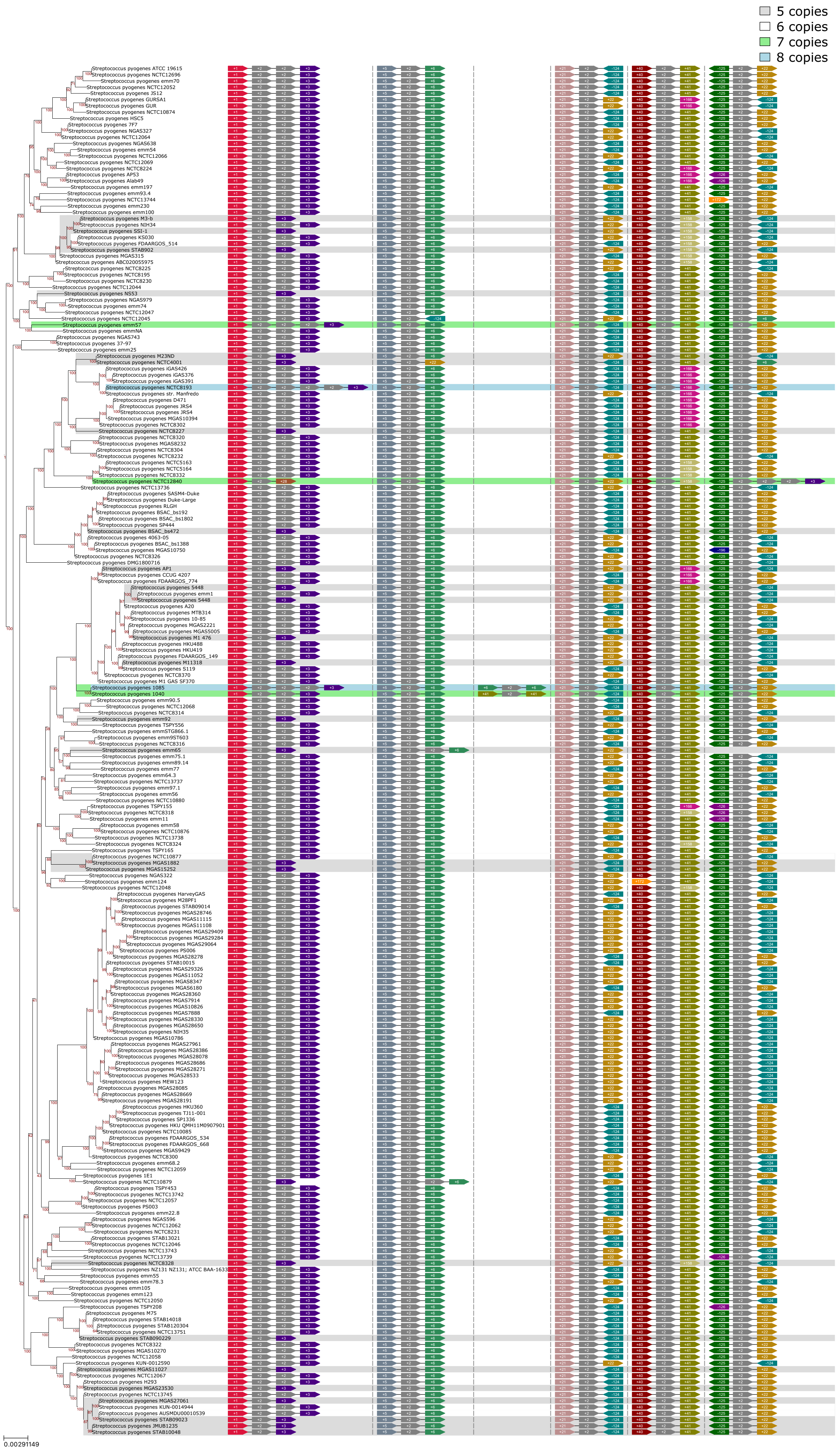

Figure S10: Genomic context of **block #2**. For each strain and each copy of the block, the upstream and the downstream neighbouring block are shown, revealing mosaic tandem duplication and numerous inversions occurred via recombination of the block's copies.

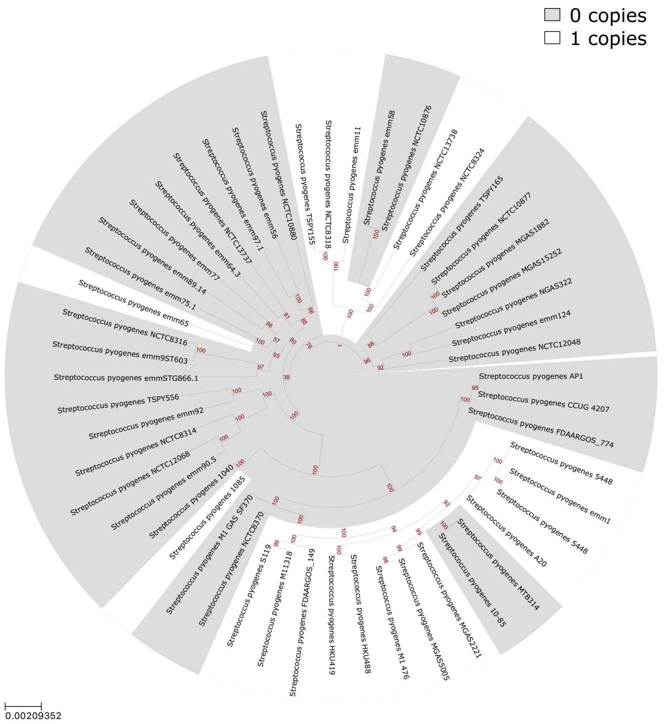

(a) Block 155

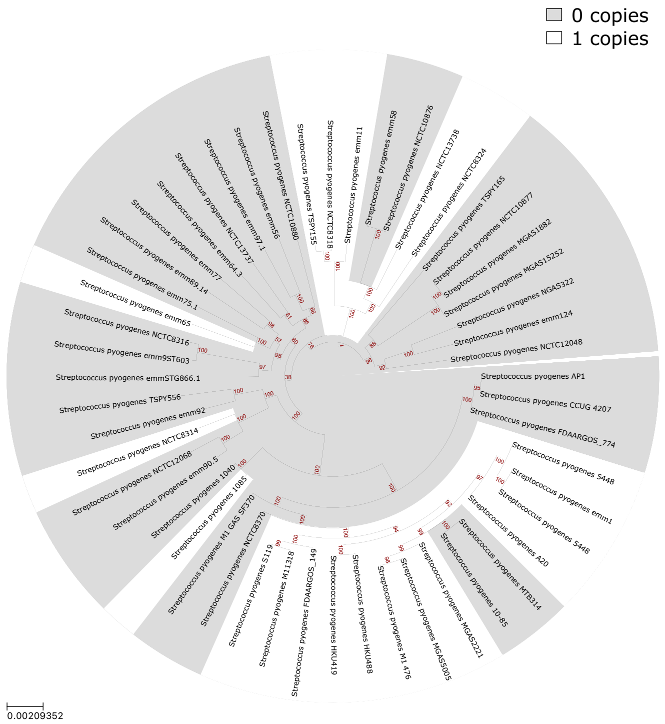

(b) Block 156

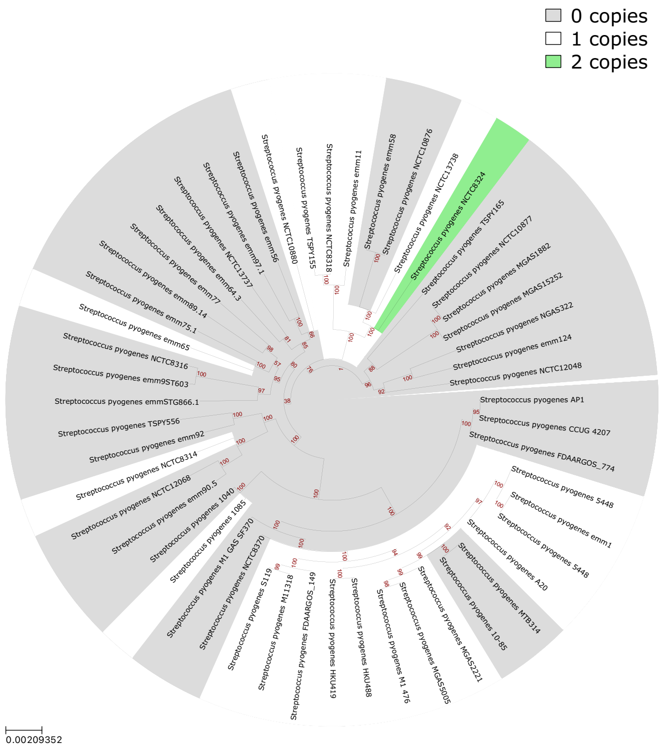

(c) Block 157

Figure S11: An example of blocks clustering, for better visibility only subtree is shown. The trees are colored to reflect the states of the characters assigned to three blocks copy number (#155, #156 and #157) placed in a row in the chromosomes. Grey state reflects the absence of this block, white and green states reflect the presence of one and two copies of this block correspondingly. PaReBrick puts them in the same cluster because these blocks are present almost in the same set of strains and placed nearly in the chromosomes. Indeed, together, these blocks form a phage insertion, some parts of which were affected by additional rearrangements in several strains.
